# The prevalence of homologous recombination deficiency (HRD) in various solid tumors and the role of HRD as a single biomarker to immune checkpoint inhibitors

**DOI:** 10.1007/s00432-021-03781-6

**Published:** 2021-09-12

**Authors:** Hana Kim, Soomin Ahn, Hongsik Kim, Jung Yong Hong, Jeeyun Lee, Se Hoon Park, Joon Oh Park, Young Suk Park, Ho Yeong Lim, Won Ki Kang, Kyoung-Mee Kim, Seung Tae Kim

**Affiliations:** 1 Division of Hematology-Oncology, Department of Medicine, Samsung Medical Center, Sungkyunkwan University School of Medicine, 81 Irwon-ro, Gangnam-gu, Seoul, 06351 Korea; 2 Department of Pathology and Translational Genomics, Samsung Medical Center, Sungkyunkwan University School of Medicine, 81 Irwon-ro, Gangnam-gu, Seoul, 06351 Korea

**Keywords:** HRD, NGS, Cancer immunotherapy, Checkpoint inhibitor

## Abstract

**Purpose:**

Homologous recombination deficiency (HRD) is related to tumorigenesis. Currently, the possibility of HRD as a prognostic biomarker to immune checkpoint inhibitors is unknown. We aimed to investigate whether HRD has potential as a biomarker for immunotherapy.

**Methods:**

The status of homologous recombination deficiency (HRD) was assessed with the next-generation sequencing (NGS) TruSight^™^ Oncology 500 assay in 501 patients with advanced solid tumor including gastrointestinal (GI), genitourinary (GU), or rare cancer. Results: among the 501 patients, HRD was observed as follows: 74.7% (347/501) patients; GU cancer (92.0%, 23 of 25), colorectal cancer (CRC) (86.1%, 130 of 151), hepatocellular carcinoma (HCC) (83.3%, 10 of 12), pancreatic cancer (PC) (76.2%, 32 of 42), biliary tract cancer (BTC) (75.0%, 36 of 48), sarcoma (65.0%, 39 of 60), melanoma (52.4%, 11 of 21), other GI cancers (50.0%, 11 of 22), and rare cancer (50.0%, 2 of 4). Sixty-five of the 501 patients had received immune checkpoint inhibitors (ICIs) during the course of the disease. Tumor types of 65 patients treated with ICIs are as follows: melanoma (95.2%, 20 of 21), HCC (33.3%, 4 of 12), rare cancer (25.0%, 1 of 4), GC (12.2%, 14 of 116), BTC (10.4%, 5 of 48), and sarcoma (5.0%, 3 of 60). The most frequently reported mutations were *BRCA2* (*n* = 90), *ARID1A* (*n* = 77), *ATM* (*n* = 71), *BARD1* (*n* = 67). Patients without HRD exhibited an objective response rate (ORR) of 33.3% (4 of 12), and patients with HRD exhibited an ORR of 34.0% (18 of 53). There was no significant difference in ORR between patients with and without HRD (*P* = 0.967). Progression-free survival (PFS) was 6.5 months (95% CI 0.000–16.175) in patients without HRD and 4.1 months (95% CI 2.062–6.138) in patients with HRD, revealing no statistical significance (*P* = 0.441).

**Conclusion:**

Herein, we reported the status of HRD using a cancer-panel for various solid tumor patients in routine clinical practice and demonstrated that HRD as a single biomarker was not sufficient to predict efficacy of ICIs in solid tumor patients.

**Supplementary Information:**

The online version contains supplementary material available at 10.1007/s00432-021-03781-6.

## Background

After immune checkpoint inhibitors (ICIs) were introduced for treatment of solid tumors, they exhibited improved survival and treatment outcomes compared to traditional non-immune anti-cancer therapies, especially for patients with advanced melanoma, non-small-cell lung cancer (NSCLC), urothelial cancer (UC), renal cell carcinoma (RCC), or other cancer types (Borghaei et al. 2015; Hodi et al. 2010; Motzer et al. 2015, 2018; Nghiem et al. 2016; Rosenberg et al. 2016; Wolchok et al. 2013). However, only some patients achieved a response to ICIs. This indicates the need for further development of immune-relevant biomarkers to identify patients who might benefit from immunotherapy.

The DNA damage repair (DDR) system is essential to maintain the integrity of the genome in organisms. Genomic alteration due to failure to repair DDR causes tumor initiation. The homologous recombination (HR) pathway has a substantial influence on genomic integrity and germline mutations in this pathway and is related to tumorigenesis (Bartkova et al. 2005; Jeggo et al. 2016; Khanna 2015). Homologous recombination is one of the major repair mechanisms of DNA double‐strand breaks. Homologous recombination deficiency (HRD) is a DNA repair deficiency related to tumorigenesis and causes increased sensitivity to platinum-based chemotherapy and PARP inhibitors (Watkins et al. 2014). The concept of therapy-directed HRD is approved in ovarian and breast cancers. The mutation in the HR pathway related to *BRCA1/2* was used to predict better objective response rates to platinum-based chemotherapy in advanced triple-negative breast cancer (Tutt et al. 2018).

Recently, targeted cancer gene panel assay or NGS for HRD has been performed in clinical settings. These panels assess genomic profiles including Tumor Mutational Burden (TMB), Microsatellite Instability (MSI), and HRD. To date, the clinical significance of gene mutations related to HRD has not been studied well across various solid tumors. Herein, we analyzed the status of HRD using cancer panels for various solid tumor patients in routine clinical practice and determined the value of HRD as a biomarker of response to ICIs.

## Methods

### Patients

Patients with pathologic confirmation of advanced gastrointestinal, GU, or rare cancers at Samsung Medical Center between Oct 2019 and Mar 2020 (*n* = 501), were prospectively tested for molecular aberrations, including TMB, with the TruSight^™^ Oncology 500 assay. All study participants provided written informed consent before study entry. The following clinicopathologic characteristics were collected for all patients: age, sex, primary tumor site, number of metastatic sites, site of metastasis, treatment, and survival. The study protocol was approved (#2020-11-151) by the Institutional Review Board of Samsung Medical Center (Seoul, Korea) and was conducted in accordance with the ethical principles of the Declaration of Helsinki and the Korea Good Clinical Practice guidelines. All patients provided written informed consent before enrollment. Written informed consent included disclosure of information, competency to make a decision, and voluntary nature of the decision for the purpose, benefit, and potential risk of this study.

### Tumor samples

Samples for analysis were collected from 501 solid tumors and prepared as formalin-fixed paraffin-embedded (FFPE) material. The samples were gathered through biopsy at diagnosis, surgical specimen, or repeat biopsy at the time of disease progression; all were obtained before immunotherapy. The types of samples used in the analysis were as follows: biopsied samples (*n* = 320, 63.9%) and surgically resected samples (*n* = 181, 36.1%).

### TruSight^™^ oncology 500assay

Forty (40) ng of DNA was quantified with the Qubit dsDNA HS Assay (Thermo Fisher Scientific) on the Qubit 2.0 Fluorometer (Thermo Fisher Scientific) and then sheared using a Covaris E220 Focused-ultrasonicator (Woburn, MA, USA) and the 8 microTUBE–50 Strip AFA Fiber V2 following the manufacturer’s instructions. Treatment time was optimized for FFPE material. The treatment settings were as follows: peak incident power (W): 75; duty factor: 15%; cycles per burst: 500; treatment time (s): 360; temperature (°C): 7; and water level: 6. For DNA library preparation and enrichment, the TruSight™ Oncology 500 Kit (Illumina) was used following the manufacturer’s instructions. Post-enriched libraries were quantified, pooled, and sequenced on a NextSeq 500 (Illumina Inc., San Diego, CA, USA). The quality of the NextSeq 500 (Illumina) sequencing runs was assessed with the Illumina Sequencing Analysis Viewer (Illumina). Sequencing data were analyzed with the TruSight^™^ Oncology 500 Local App Version 1.3.0.39 (Illumina), a comprehensive tumor profiling assay designed to identify known and emerging tumor biomarkers, including small variants, splice variants, and fusions. The reads were aligned to the reference genome (GRCh37/hg19) using Burrows − Wheeler Aligner-MEM (BWA-MEM) (Li 2013). Poorly mapped reads with a mapping quality (MAPQ) below 20 were removed using Samtools version 1.3.1(Li et al. 2009). Somatic mutations including single-nucleotide variants (SNV) and small insertions and deletions (INDELs) were detected by the Pisces and Psara (Dunn et al. 2019). The rest of pipeline are as follows: CRAFT for copy number variation, TmbRaider for TMB, Hubble for MSI, STAR for RNA alignment, and Manta for fusion calling (Pestinger et al. 2020). Outputs of data, exported from The TSO 500 pipeline (Pestinger et al. 2020) were annotated with Ensembl Variant Effect Predictor (VEP) Annotation Engine, with information from the databases, such as dbSNP, gnomAD genome and exome, 1000 genomes, ClinVar, COSMIC, RefSeq, and Ensembl. The processed genomic alterations were categorized with four-tier system by American Society of Clinical Oncology and College of American Pathologists (Li et al. 2017), annotated with proper reference. The following criteria were used to filter our less significant variants and possible germline variants: (i) variants < 5% allele frequency and < 100 × read depth at the variant were excluded; (ii) variants previously reported to be benign or likely benign in the ClinVar archive (Landrum et al. 2016) were excluded; (iii) variant with a frequency greater than 1% in gnomAD (Karczewski et al. 2020) were excluded.

Importantly, the TruSight^™^ Oncology 500 measures homologous recombination deficiency (HRD). The HRD-related genes were as follows: *ARID1A, ATM, ATRX, BAP1, BARD1, BLM, BRCA1, BRCA2, BRIP1, CHEK1, CHEK2, FANCA, FANCC, FANCD2, FANCE, FANCF, FANCG, FANCL, MRE11A, NBN, PALB2, PTEN, RAD50, RAD51,* and *RAD51B.* Homologous recombination deficiency was diagnosed if there was at least one HR-related gene mutation.

### Statistical analyses and disease evaluation

All statistical analyses were conducted with SPSS statistics 27. Descriptive statistics are reported as proportion and median. Data are presented as number (%) for categorical variables. Correlations between status of HRD and clinicopathologic features were analyzed by *t* test, Fisher’s exact test, or one-way analysis of variance (ANOVA), as appropriate. Response categories were assessed according to RECIST 1.1 (Eisenhauer et al. 2009; Schwartz et al. 2016). Objective response rate (ORR) was defined as the percentage of patients with complete response (CR) or partial response (PR). Progression-free survival (PFS) was defined as the interval between the initiation of the treatment and the time of progressive disease (PD). Logistic regression analysis was performed to analyze HRD genes that might be related to treatment response. A Cox regression model was used to analyze the associations of suspecting factors, including HRD and disease progression after ICIs treatment. The Mann–Whitney test was used to compare the difference between HRD and non-HRD. Kaplan–Meier estimates and log-rank tests were used in analysis of all time to event variables, and 95% confidence interval for the median time to event was computed.

## Results

### Patient characteristics

**Fig. 1:**
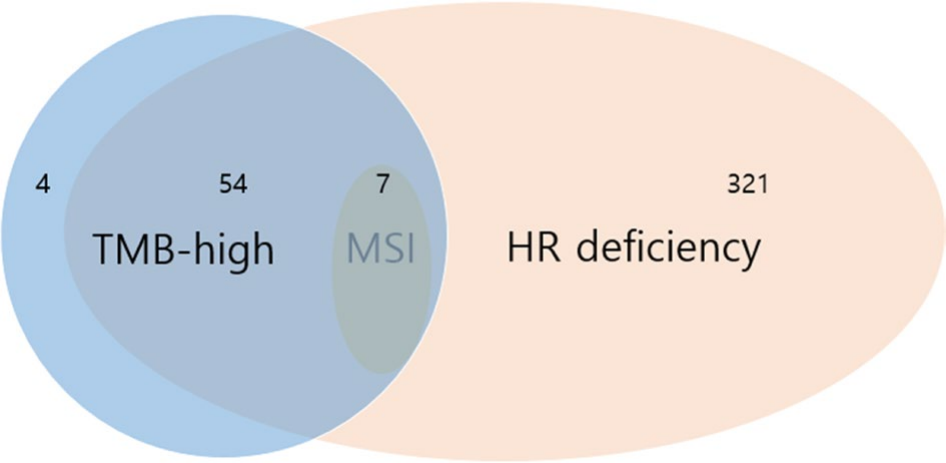
Distributions of TMB-high, MSI-high, and HRD analyzed by NGS panel in various solid tumors. *TMB* tumor mutational burden, *MSI* microsatellite instability, *HRD* homologous recombination deficiency

Table 1 presents the clinical characteristics of the 501 patients included in this study. The median age of the patients was 59.7 years (range, 21–86), and the majority were male (60.3%). The median age of males was 61 years, while that of female was 58 years. The most frequent tumor type was colorectal cancer (*n* = 151, 30.1%), followed by gastric cancer (*n* = 116, 23.2%), sarcoma (*n* = 60, 12.0%), pancreatic cancer (*n* = 42, 8.4%), genitourinary (GU) cancer (*n* = 25, 5.0%), other gastrointestinal (GI) tract cancer (*n* = 22, 4.4%), melanoma (*n* = 21, 4.2%), hepatocellular carcinoma (HCC) (*n* = 12, 2.4%), and rare cancer (*n* = 4, 0.8%). Among the 501 patients, 65 had been treated with immune checkpoint inhibitors (ICIs). Figure 1 shows the distribution of TMB, MSI, and HR deficiencies. All seven patients with MSI were TMB-high and HR-deficient. On the other hand, of 375 patients with HR deficiency, only 54 were confirmed to be TMB-high or MSI.

**Table 1:** Characteristics of 501 patients with various solid tumors

	All patients (*N* = 501)
Age (year)	
Median (range)	59.7 (21–86)
Sex	
Male	302 (60.3%)
Female	199 (39.7%)
Tumor type	
Colorectal cancer	151
Gastric cancer	116
Sarcoma	60
Biliary tract cancer	48
Pancreatic cancer	42
Genitourinary cancer	25
Other GI tract cancer	22
Melanoma	21
Hepatocellular carcinoma	12
Rare cancer	4
Tumor mutational burden (TMB)	
TMB low	443 (88.4%)
TMB-high	58 (11.6%)
Microsatellite instability (MSI)	
Non-MSI	494 (98.6)
MSI	7 (1.4)
PD-L1 (*N* = 225)	
Positive	101 (20.2)
Negative	124 (24.8)
Homologous recombination (HR)	
Deficiency	375 (74.9%)
Non-deficiency	126 (25.1%)
Receiving ICIs	
Yes	65 (13.0%)
No	436 (87.0%)

### Frequency of tumors with HRD according to type

**Fig. 2:**
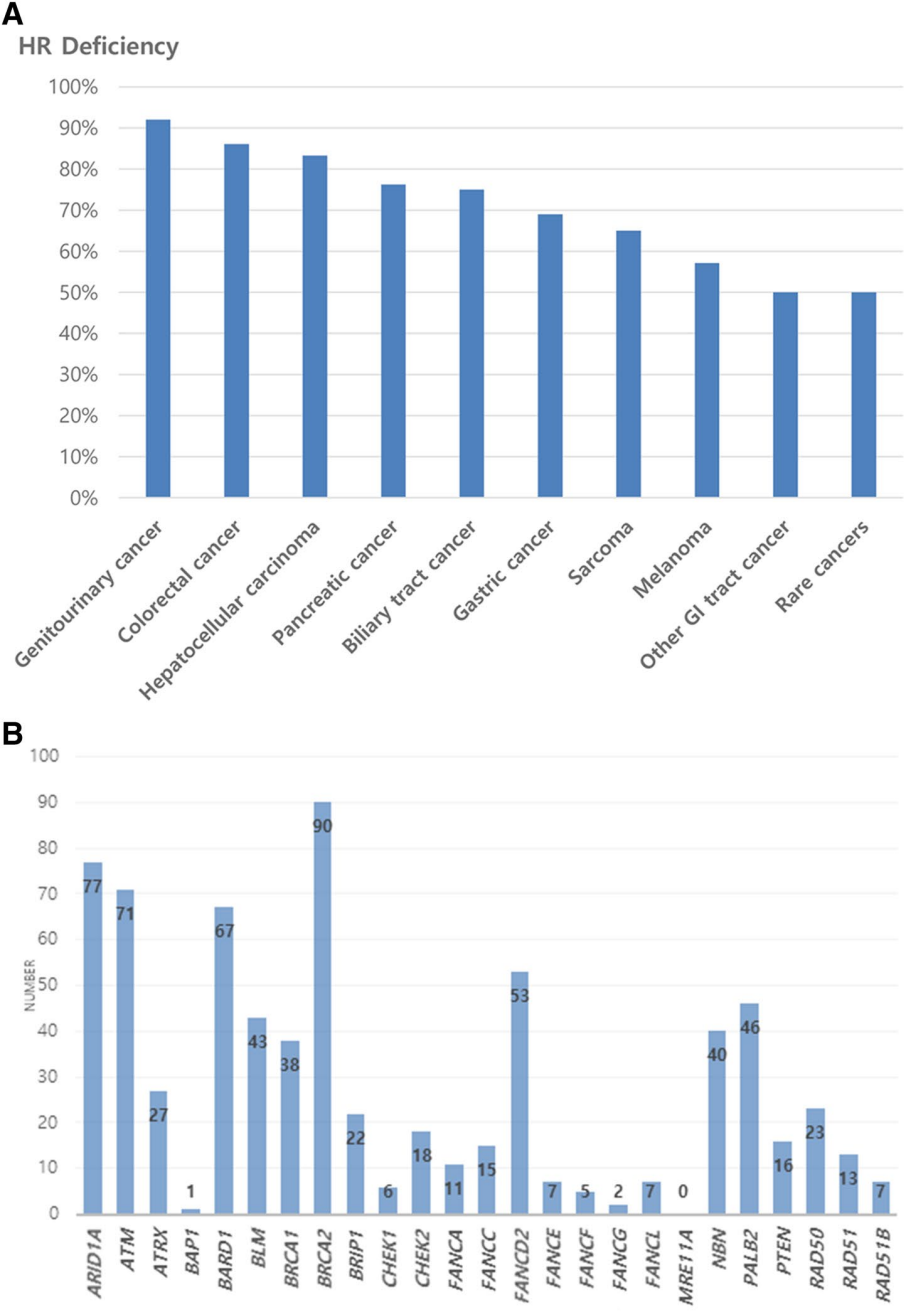
Prevalence of homologous recombination deficiency (HRD) and HR-related gene mutations in various solid tumors by NGS TruSight. **A** HRD prevalence by various tumor types in order of highest ratio. **B** The observed frequency of HR-related gene variations by NGS panel **A**, **B**

Tumors with HRD were observed in 375 of 501 patients irrespective of type. Table 2 presents the status of the HRD and the ratio of patients who received ICI treatment according to tumor type. HR deficiency was observed in 74.9% of patients with various solid tumors including GU cancer (92.0%, 23 of 25), CRC (86.1%, 130 of 151), HCC (83.3%, 10 of 12), pancreatic cancer (76.2%, 32 of 42), biliary tract cancer (75.0%, 36 of 48), gastric cancer (69.0%, 80 of 116), sarcoma (65.0%, 39 of 60), melanoma (57.1%, 12 of 21), other GI tract cancer (AOV cancer, appendiceal cancer, cecal cancer, duodenal cancer, GIST) (50.0%, 11 of 22), and rare cancer (50.0%, 2 of 4). Figure 1 presents the distribution relationship with other biomarkers. All MSI were TMB-high and HR-deficient. However, some TMB-high have no HR deficiency. Figure 2A shows the percentage of confirmed HRD for each tumor type listed in order of high frequency rate. The tumor with the highest frequency of HRD was GU cancer with 92.0% and the lowest frequency was other GI tract cancer (AOV cancer, appendiceal cancer, cecal cancer, duodenal cancer, and GIST) and rare cancer at 50.0%. The distribution of HRD mutations for each cancer type is included in the supplement. (Supplement S1).

**Table 2:** Prevalence of homologous recombination deficiency (HRD) and use of immune check point inhibitors (ICIs) according to tumor type

Tumor type	HR deficiency	ICIs
Colorectal cancer (151)	130 (86.1%)	4 (2.6%)
Gastric cancer (116)	80 (69.0%)	14 (12.1%)
Sarcoma (60)	39 (65.0%)	3 (5.0%)
Biliary tract cancer (48)	36 (75.0%)	5 (10.4%)
Pancreatic cancer (42)	32 (76.2%)	1 (2.4%)
Genitourinary cancer (25)	23 (92.0%)	12 (48.0%)
Other GI tract cancer^a^ (22)	11 (50.0%)	1 (4.5%)
Melanoma (21)	12 (57.1%)	20 (95.2%)
Hepatocellular carcinoma (12)	10 (83.3%)	4 (33.3%)
Rare cancer^b^ (4)	2 (50.0%)	1 (25.0%)
Total 501	375 (74.9%)	65 (13.0%)

^a^AOV cancer, appendiceal cancer, cecal cancer, duodenal cancer, and GIST

^b^Adrenocortical cancer and MUO (malignancy of unknown primary)

### Frequency of HRD according to HR-related genes

We also analyzed the observed genetic variations by HR-related genes. (Fig. 2B) The most frequently reported mutations were *BRCA2* (*n* = 90), *ARID1A* (*n* = 77), *ATM* (*n* = 71), *BARD1* (*n* = 67). On the other hand, *FANCG* was observed twice and *BAP1* gene was reported only once. Even *MRE11A* gene was never observed in 501 patients’ NGS results.

### Correlations between HRD and disease progression in 65 patients treated with ICIs

Sixty-five patients treated with ICIs at diagnoses as follows: melanoma (95.2%, 20 of 21), HCC (33.3%, 4 of 12), rare cancer (25.0%, 1 of 4), GC (12.2%, 14 of 116), BTC (10.4%, 5 of 48), and sarcoma (5.0%, 3 of 60) (Table 2). We analyzed the correlation between HRD and efficacy to ICIs. Patients with HRD exhibited an objective response rate (ORR) of 27.3% (3 of 11), while patients without HRD achieved an ORR of 39.0% (16 of 41).

**Fig. 3:**
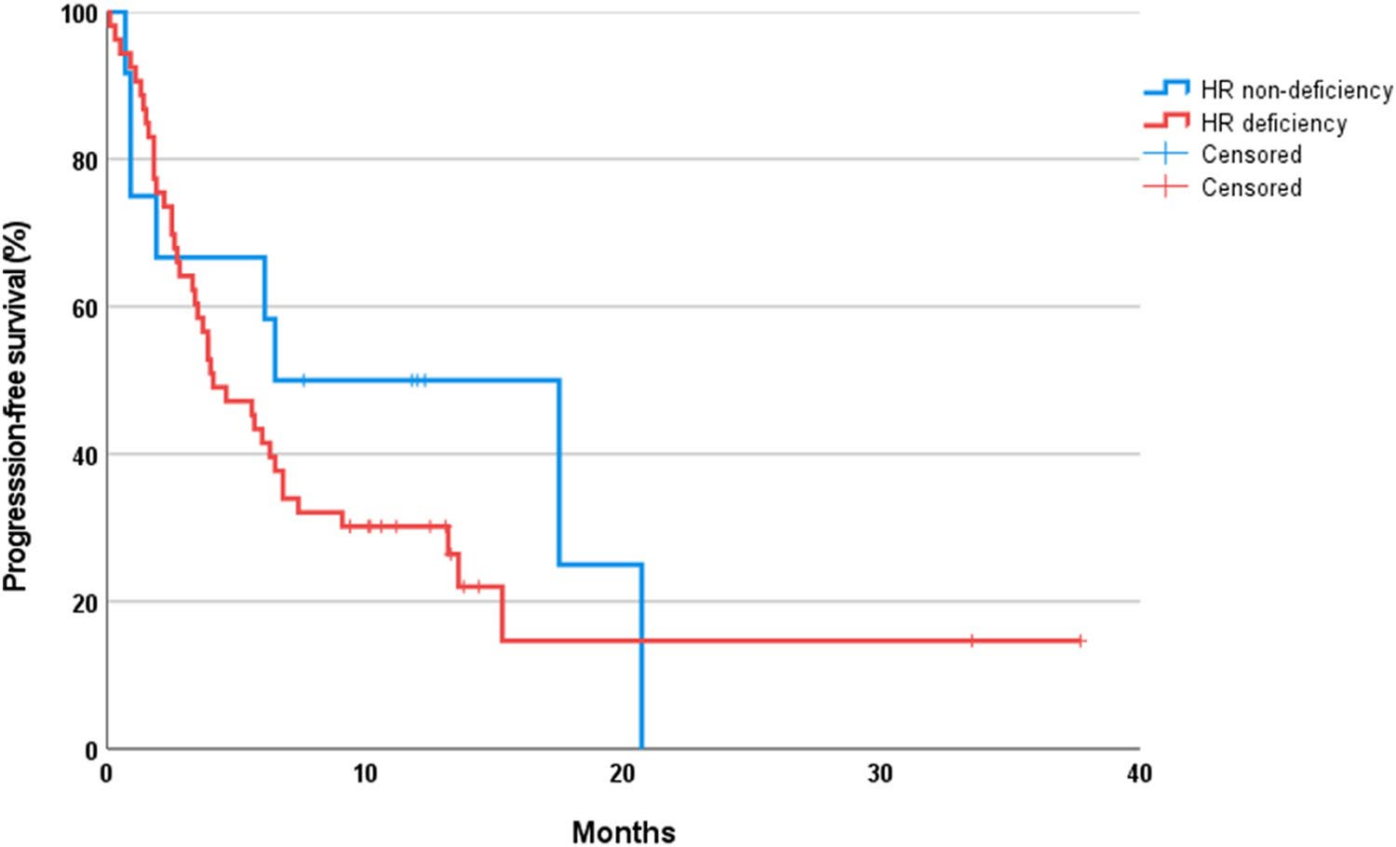
Kaplan–Meier curve for Progression-free survival (PFS) after immune checkpoint inhibitors according to homologous recombination deficiency (HRD) status, (*N* = 65)

Progression-free survival (PFS) after ICIs was 6.5 months (95% CI 0.000–16.175) in patients without HRD and 4.1 months (95% CI 2.062–6.138) in patients with HRD. This difference was not significant (*P* = 0.441) (Fig. 3). Detailed data on HRD status and progression for each cancer type are included in the supplement (Supplement S2).

Additionally, we conducted Cox proportional hazard analysis for PFS after ICIs (Table 3). TMB was the only meaningful prognostic factor (*P* = 0.019). Response after immunotherapy was analyzed logistic regression and only TMB was revealed to be statistically significant (*P* = 0.004). It has been previously reported in our study that TMB by NGS panel is a useful predictor of immunotherapy (Kim et al. 2021).

**Table 3:** Analyses for risk factors affecting progression free survival (PFS) by Cox proportional hazard ratio and objective response to immunotherapy by logistic regression model

Variables	Cases	PFS	
		OR (95.0% CI)	*P*
Age			
< 65	37		
≥ 65	28	0.707 (0.394–1.267)	0.244
Smoking			
No	38		
Yes	27	1.037 (0.584–1.842)	0.900
HRD			
0 (non-deficiency)	12		
1 (deficiency)	53	1.376 (0.639–2.963)	0.415
TMB			
Low	50		
High	15	0.396 (0.182–0.859)	0.019
Microsatellite instability			
Non-MSI	61		
MSI	4	0.357 (0.086–1.486)	0.157
PD-L1 by IHC			
Negative	16		
Positive	15	1.000 (0.999–1.000)	0.378

Variables	Cases	Response to ICIs	
		Exp(β) (95.0% CI)	*P*
Age			
< 65	37		
≥ 65	28	1.157 (04,411–3.258)	0.782
Smoking			
No	38		
Yes	27	0.722 (0.251–2.077)	0.545
HRD			
0 (non-deficiency)	12		
1 (deficiency)	53	1.029 (0.273–3.881)	0.967
TMB			
Low	50		
High	15	6.333 (1.806–22.204)	0.004
Microsatellite instability			
Non-MSI	61		
MSI	4	6.632 (0.647–67.964)	0.111
PD-L1 by IHC			
Negative	16		
Positive	15	1.000 (0.999–1.001)	0.431

*CI* confidence interval, *OR* odds ratio*, Exp(β*) exponentiated coefficient, ICIs immune checkpoint inhibitors

## Discussion

In the present study, we evaluated the prevalence of HRD in 501 patients with various solid tumors and investigated the role of HRD as a single biomarker to predict response to ICIs. The overall prevalence of HRD we analyzed was 74.7% (347/501) and especially, GU cancer and CRC had the HRD of the high frequency. In 65 patients with ICIs, there were no significant differences for ORR and PFS between patients with and without HRD (*P* = 0.967 and *P* = 0.441, respectively). These findings suggested that HRD as a single biomarker was not sufficient to predict the efficacy of ICIs in solid tumor patients.

The overall frequency of HRD we analyzed was 74.7% (347/501). This finding was not consistent with other studies about HRD. A previous study reported that the prevalence of HR-DDR mutations was 17.4% in multiple tumor types (Heeke et al. 2018). This discordance might be caused by different studied genes including different NGS panels and different genes defining HRD. Detection of HRD by the NGS panel has limitations. There is no established definition to assess HRD. Therefore, there are many different results among published papers about the prevalence of HRD. Furthermore, this difference might be caused by discrepancy between measurement of HRD with whole exome sequencing and NGS panels.

There are a few limitations to this study. First, it was a retrospective study, and clinically heterogeneous populations were subject to potential biases. Second, only the Asian population was assessed in the study, so differences in genomic profiles and clinical features between Western and Eastern patients with solid tumors were not considered. Also, this study included a relatively small proportion of patients who had been treated with ICIs, making it difficult to draw definite conclusions regarding biomarkers.

To assess HRD, loss heterozygosity, number of telomeric allelic imbalance and large-scale state transitions are needed (Konstantinopoulos et al. 2015; Patel et al. 2018). However, these parameters are not available in TSO 500 but instead provide point mutations of HR-related genes. Detecting point mutations in HR genes using DNA sequencing panels to identify HR-deficient tumor is previously described (Pellegrino et al. 2020; Polak et al. 2017). In a study with renal cell carcinoma, mutation in HR-related gene associated with higher mutation burden in association with disease control (Labriola et al. 2020) and germline or somatic mutation of *BRCA* were associate with high mutational burden and showed different genetic character in breast cancer (Lal et al. 2019). The clinical significance of mutation in HR-related genes for application in immunotherapy still needs further investigation with larger cohort and sufficient follow-up period. In addition, future studies on the selection and cut-off value for HR-related gene numbers are also expected, as a biomarker development.

HRD might be a potential candidate predictor of response to ICIs, but the prevalence of HRD has not been investigated across tumor types. The present analysis produced useful information on the prevalence of HRD in various solid tumors under routine clinical practice and demonstrated that HRD as a single biomarker was not sufficient to predict the efficacy of ICIs in solid tumor patients.

## Conclusion

Herein, we reported the status of HRD using a cancer panel for various solid tumor patients in routine clinical practice and demonstrated that HRD as a single biomarker was not sufficient to predict efficacy of ICIs in solid tumor patients.

## Supplementary Information

Below is the link to the electronic supplementary material.

Supplementary file1 (PDF 299 KB)

## Data Availability

All data that can prove the conclusion of this article are included in the article and the supplements.

## Abbreviations

BTC: Biliary tract cancer
CRC: Colorectal cancer
DDR: DNA damage repair
FFPE: Formalin-fixed paraffin-embedded
GU: Genitourinary
HCC: Hepatocellular carcinoma
HRD: Homologous recombination deficiency
ICIs: Immune checkpoint inhibitors
MSI: Microsatellite instability
NGS: PC: Pancreatic cancer next-generation sequencing
ORR: Objective response rate
PFS: Progression-free survival
RCC: Renal cell carcinoma
TMB: Tumor mutational burden
