# Supplementary material for: The prevalence of homologous recombination deficiency (HRD) in various solid tumors and the role of HRD as a single biomarker to immune checkpoint inhibitors
Source: J Cancer Res Clin Oncol. 2021 Sep 12;148(9):2427–35. doi: 10.1007/s00432-021-03781-6 (PMC9349061; doi:10.1007/s00432-021-03781-6)
Supplement: Supplementary file 1 — Supplementary file1 (PDF 299 KB) [file 432_2021_3781_MOESM1_ESM.pdf]

S1. The distribution of HRD mutations for each cancer type

	Colo-rectal cancer (n=151)	Gastric cancer (n=116)	Sarcoma (n=60)	Biliary tract cancer (n=48)	Pancreatic cancer (n=42)	Genitourinary cancer (n=25)	Other GI cancer (n=22)	Melanoma (n=23)	HCC (n=10)	Rare cancer (n=4)	Total
**ARID1A**	24	19	6	9	7	4	4	2	2	0	77
**ATM**	25	13	4	6	9	4	3	4	2	1	71
**ATRX**	9	3	5	2	4	2	0	1	0	1	27
**BAP1**	0	0	0	0	0	0	0	1	0	0	1
**BARD1**	24	11	11	4	6	5	1	3	1	1	67
**BLM**	16	6	5	3	6	3	4	0	0	0	43
**BRCA1**	11	5	3	5	3	5	2	3	0	1	38
**BRCA2**	26	20	9	14	9	7	3	1	1	0	90
**BRIP1**	5	4	4	2	2	2	2	1	0	0	22
**CHEK1**	3	1	0	2	0	0	0	0	0	0	6
**CHEK2**	6	6	0	1	1	2	0	1	1	0	18
**FANCA**	4	5	1	1	0	0	0	0	0	0	11
**FANCC**	7	3	0	2	0	0	2	0	1	0	15
**FANCD2**	26	8	6	3	2	4	1	0	3	0	53
**FANCE**	3	2	0	0	0	1	0	1	0	0	7
**FANCF**	2	0	1	1	0	0	0	0	1	0	5
**FANCG**	0	2	0	0	0	0	0	0	0	0	2
**FANCL**	2	2	1	1	0	0	1	0	0	0	7
**MRE11A**	0	0	0	0	0	0	0	0	0	0	0
**NBN**	11	14	5	4	1	3	0	0	1	1	40
**PALB2**	13	9	3	3	1	2	3	0	2	0	46
**PTEN**	8	3	1	2	0	2	0	0	0	0	16
**RAD50**	12	4	1	2	1	2	0	1	0	0	23
**RAD51**	6	2	1	2	1	0	0	0	1	0	13
**RAD51B**	3	0	2	1	0	0	1	0	0	0	7

S2-A HRD and progression status for each cancer type

Tumor type	Total (N = 65)	Progression (n)	HRD mutation	HRD mutation with progression
Colorectal cancer	4	2	4	2
Gastric cancer	14	12	12	10
Sarcoma	3	2	2	2
Biliary tract	5	4	5	4
Pancreatic	1	1	1	1
Genitourinary	12	8	12	8
Other GI cancer	1	1	1	1
Melanoma	20	14	11	8
Hepatocellular carcinoma	4	3	4	3
Rare cancer	1	1	1	1

GI gastrointestinal, HRD Homologous recombination deficiency

S2-B Kaplan-Meier curves for progression-free survival of each cancer type according to HRD status.

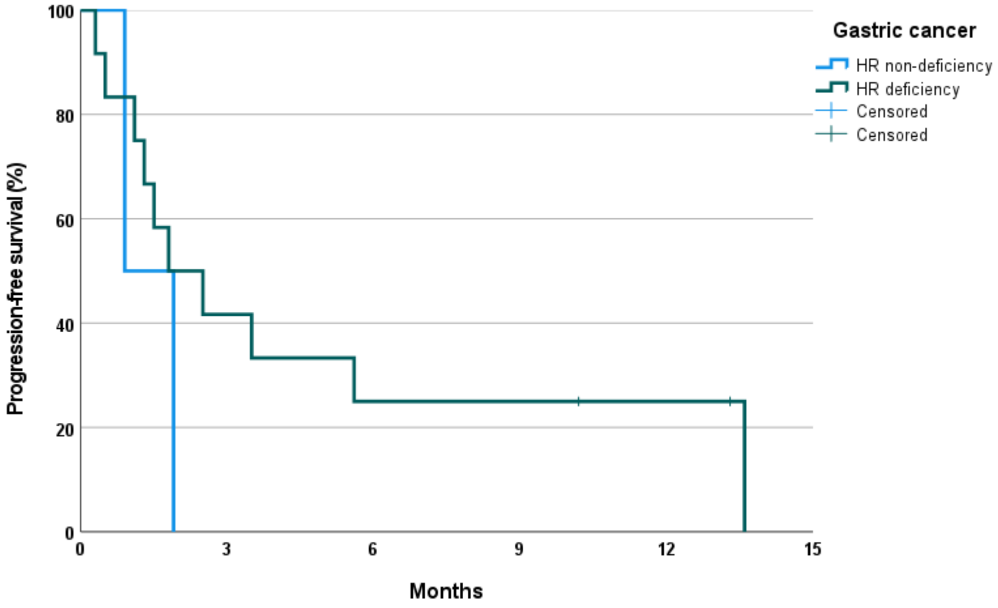

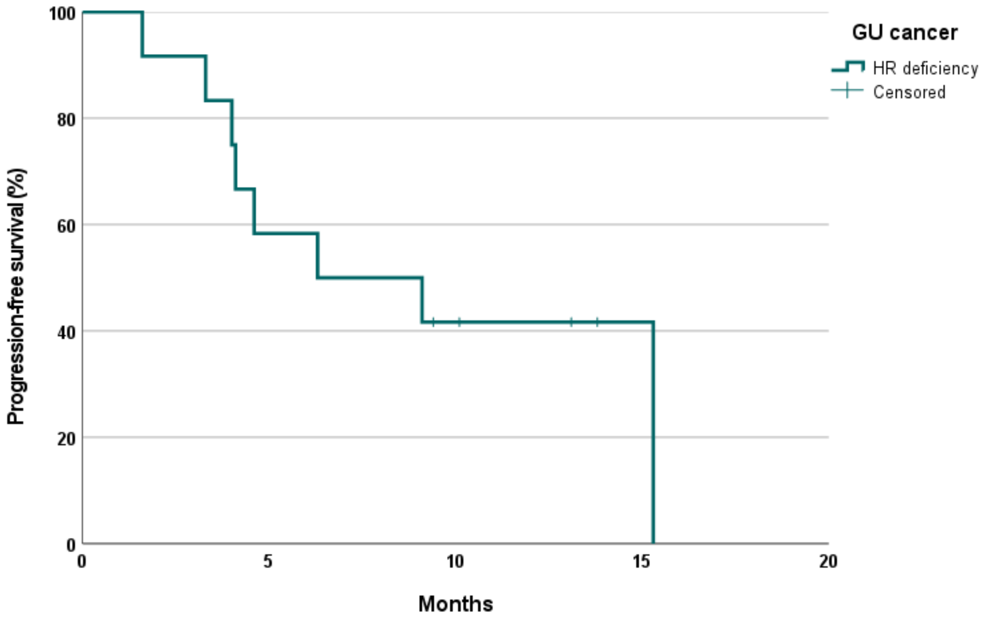

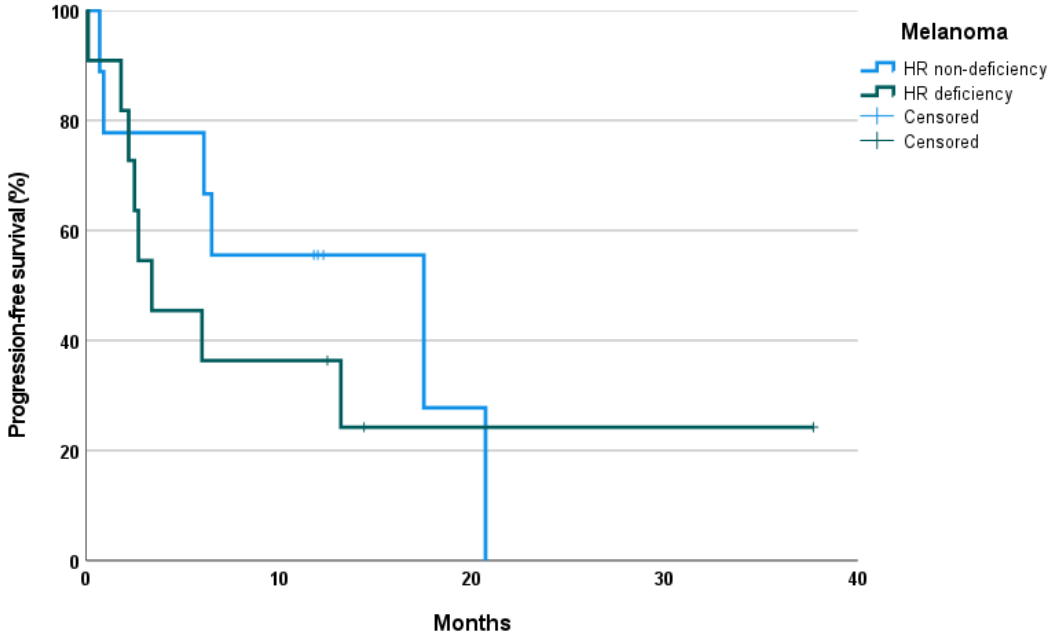

GU genitourinary
